# A first-principles analysis of the charge transfer in magnesium corrosion

**DOI:** 10.1038/s41598-020-71694-4

**Published:** 2020-09-14

**Authors:** Tim Würger, Christian Feiler, Gregor B. Vonbun-Feldbauer, Mikhail L. Zheludkevich, Robert H. Meißner

**Affiliations:** 1 Institute of Materials Research, Helmholtz-Zentrum Geesthacht Centre for Materials and Coastal Research, Geesthacht, Germany; 2 Institute of Polymer and Composites, Hamburg University of Technology, Hamburg, Germany; 3 Institute of Advanced Ceramics, Hamburg University of Technology, Hamburg, Germany; 4 Institute for Materials Science, Faculty of Engineering, University of Kiel, Kiel, Germany

**Keywords:** Materials chemistry, Computational chemistry, Density functional theory, Electrochemistry, Corrosion

## Abstract

Magnesium is the lightest structural engineering material and bears high potential to manufacture automotive components, medical implants and energy storage systems. However, the practical use of untreated magnesium alloys is restricted as they are prone to corrosion. An essential prerequisite for the control or prevention of the degradation process is a deeper understanding of the underlying corrosion mechanisms. Prior investigations of the formation of gaseous hydrogen during the corrosion of magnesium indicated that the predominant mechanism for this process follows the Volmer–Heyrovský rather than the previously assumed Volmer–Tafel pathway. However, the energetic and electronic states of both reaction paths as well as the charge state of dissolved magnesium have not been fully unraveled yet. In this study, density functional theory calculations were employed to determine these parameters for the Volmer, Tafel and Heyrovský steps to gain a comprehensive understanding of the major corrosion mechanisms responsible for the degradation of magnesium.

## Introduction

Light-weight materials with outstanding mechanical properties as well as novel anode materials for energy storage systems represent promising strategies to combat some of the adverse impacts caused by the ongoing climate change^1,2^. Concomitantly, modern engineering materials are not only required to excel in a single discipline but are rather expected to stand out in a multitude of properties in order to meet the high standards of pioneering industrial applications. Treated magnesium-based alloys are promising candidates to satisfy a variety of current demands of the automotive industry^3^ as well as concerning medical^4^, and battery applications^5,6^.

However, in all of those research areas corrosion plays a key role and thus renders measures to control degradation processes in the employed material vital for its implementation. There are several approaches to protect magnesium from corrosion, including alloying and surface coatings^7,8^. Furthermore, the introduction of magnesium dissolution modulators^9–13^, chemical compounds that inhibit or promote magnesium corrosion, facilitates tuning of the corrosion rate to meet application-specific demands. The latter approach allows for tailored degradation rates of resolvable medical implants (e.g. stents) or adjustment of properties of the anode material in magnesium-air batteries to achieve high utilization efficiencies while maintaining a high discharge potential. However, the implementation of these approaches in magnesium engineering could be greatly enhanced by profound knowledge of the underlying corrosion mechanisms (e.g. evolution of hydrogen) which are still not fully understood to date. In aqueous environments, the overall corrosion reaction is given as^14^:1consisting of oxidation of magnesium and water reduction, described by the partial reactions Eqs. (2) and (3), respectively:23Although the oxygen reduction reaction (ORR), given by4also occurs as a cathodic process and contributes to the overall corrosion reaction in Eq. (1), its contribution is relatively small for high corrosion rates and is thus neglectable. Hence, the water reduction reaction described in Eq. (3) dominates the corrosion process and is considered here as major cathodic reaction^14,15^.

Magnesium hydroxide emerges due to a recombination of dissolved magnesium ions with previously formed hydroxide ions:5The hydrogen evolution (HE) pathway is initiated by water reduction in the Volmer step, followed by hydrogen adsorption on the magnesium surface.6Subsequently, the HE process can continue in two possible ways, described by Eqs. (7) and (8): the adsorbed hydrogen atom either recombines with another adsorbed hydrogen atom in the homolytic Tafel step, or reacts directly with the partially positive hydrogen atom of a close water molecule in the heterolytic Heyrovský step^16–19^:78The energetics and kinetics of the hydrogen evolution reactions (HERs) on Mg(0001) were already discussed in the literature^20–24^. In a theoretical study by Yuwono et al., the Volmer–Heyrovský pathway was found to be energetically more favorable than the Volmer–Tafel pathway, and thus, to largely contribute to the HE at cathodic and anodic overpotentials^21^. However, the reaction in Eq. (8) requires excess electrons. In a study by Surendralal et al., the authors found that the adsorbed hydrogen atom is a hydride ($\hbox {H}^-$)^25^ and not in a charge neutral state as previously presumed^26^. Thus, they proposed a “Heyrovský-like” reaction9where the excess electron required for Eq. (8) is provided by the adsorbed hydride itself. This is in good agreement with studies on Mg-based alloys and hydrogen storage systems^27,28^ that also observed adsorption of hydrides on the used substrate. However, a detailed investigation of the charge transfer during HE on the magnesium surface has not been conducted yet and detailed studies on an atomistic level will facilitate fully unraveling the reaction mechanisms accompanying Mg corrosion.

As experimental approaches often require complementary information to explain the occurring corrosion mechanisms on an atomistic level in detail, quantum mechanical computations constitute a highly promising strategy to gain deeper insight into the fundamental electrochemical reactions of Mg corrosion in an aqueous environment^21,22,29^. However, the physical concept behind magnesium corrosion is not determined by a single mechanism, but rather resembles an interplay between different factors such as corrosion product formation^30^, anodic regions^31^ and noble impurities^32^. Also the formation of an $\hbox {Mg}^{+}$ species which promotes water reduction is a constant topic of discussion^14,33,34^. Consequently, establishing an accurate computational model that incorporates all, or at least most, of the inherent factors that influence the corrosion of Mg is highly complex. In particular, the atomistic simulation of electrified solid/liquid interfaces is still highly challenging and lacks simple and intuitive approaches. Although recently developed techniques to combine electrochemistry and thermodynamics in atomistic simulations allow to actively relate quantum mechanical results with electrochemical parameters, such as voltage and pH^16,18,20^ , this still remains a challenging task. Additionally, sophisticated quantum mechanical calculations are limited to rather small system sizes of a few hundred atoms due to high computational costs and should optimally be validated by experimental results. Nevertheless, the quantum mechanical *ab initio* methods performed in this study have high potential regarding the modeling of fragments of the Mg corrosion process as they provide new insights into the underlying electrochemical mechanisms, particularly addressing reaction kinetics as well as energetic states and electronic charge distributions.

## Results

**Figure 1:**
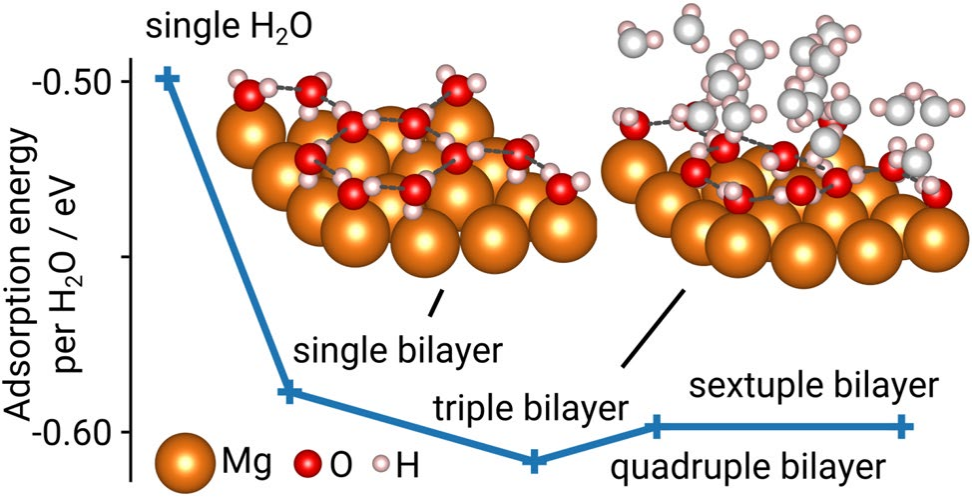
Influence of the amount of stacked water bilayers on the interface structure and the adsorption energy per $\hbox {H}_2\hbox {O}$ molecule. Water molecules not directly interacting with the Mg(0001) surface are depicted as gray. The ice-like hexagonal water bilayer structure at the solid–liquid interface is indicated by dashed lines. The results indicate that a single water bilayer describes the adsorption energy sufficiently as additional bilayers exhibit an insignificant impact on the calculated energies.

Several structural aspects have to be elucidated prior to developing a representative system for the hydrogen evolution reactions (HER) during aqueous Mg corrosion. Firstly, water plays a major role in the primary reaction mechanisms—namely water dissociation (Volmer pathway), hydrogen evolution (via the Tafel or Heyrovský pathway) and magnesium dissolution. The aqueous environment can be modeled in different ways, including implicit solvent models^24^ as well as the addition of explicit water molecules^21^. In accordance with the literature^21,35,36^, in DFT calculations ice-like hexagonal water bilayers were found to be most stable configuration at the magnesium-water interface for a surface saturated with $\hbox {H}_2\hbox {O}$. Here, every second water molecule binds with its oxygen molecule to the top site of the magnesium surface at a distance of 2.16 Å. The other water molecules may adopt two possible orientations, referred to as H-down or H-up structures, with one hydrogen atom either pointing towards or away from the surface, respectively^35^. With a calculated energy difference $\Delta E_{{\mathrm{up}}{-}{\mathrm{down}}}=0.05$ eV, the H-down configuration is energetically slightly more favorable for a single bilayer (consisting of six explicit water molecules) whereas for multiple water bilayers the system relaxed to a distorted mixture of both configurations due to interlayer hydrogen bonds. Despite a growing hydrogen bond network for additional water bilayers which also influences molecular orientations at the magnesium-water interface, the adsorption energies per $\hbox {H}_2\hbox {O}$ molecule remain mostly constant ($E_{{\mathrm{ads,H}}_2{\mathrm{O}}}=0.60\pm 0.01$ eV) after full saturation of the Mg(0001) surface (see Fig. 1).

However, simulations of the Volmer–Tafel and Volmer–Heyrovský pathways on the solvated Mg(0001) surface revealed that a varying number of water bilayers has an influence on the energy of formation $\Delta E_{\mathrm{form}}$ as well as the reaction barriers (see Supplementary Table S3). As shifts in the water structure can evoke dominating changes in the energetic landscapes of the analyzed reaction pathways, simulations including a single bilayer are analyzed in more detail in the following with the exception of the models concerning the magnesium dissolution reaction pathway. In this case four bilayers were used to ensure a sufficient amount of water for a full solvation shell around the magnesium ion.

Additional prerequisites to model an electrochemical reaction in a climbing image nudged elastic band (NEB) simulation are that products and reactants are known as well as an intuition for the potential reaction pathway. Hence, energetically favorable adsorption sites for the reactants during HER have to be identified. As experiments at corroding Mg surfaces indicate low concentrations of H_3_O^+^ in a magnitude of 10^−12^ M but significantly higher concentrations of around 55 M H_2_O, the presence of H_3_O^+^ ions and their influence on the energy barriers is assumed not to be significant for real conditions and thus neglected in the following simulations. Although the adsorption energetics of the considered reactants ($\hbox {H}_2\hbox {O}$, $\hbox {OH}^{-}$ and $\hbox {H}^{+}$) have already been investigated in detail^23^, the computations were replicated using the optB88-vdW functional as the impact of dispersion effects was neglected in the study of Williams et al. (for details see Supplementary Tables S1–S3) and other binding sites might become more favorable taking dispersion into account.

Analogously to Yuwono et al.^20,21^ the simulation conditions of the underlying model system correspond to a pH 0 at open circuit potential (OCP). Only the major reactants are included in the simulations of the reaction pathways and potentially co-adsorbed hydroxide ions are neglected as the adsorption of $\hbox {OH}^{-}$—e.g. from a preceding Volmer step—can induce a local pH change to more alkaline conditions, thus influencing the calculated energy barriers. For example, higher pH values generally decrease the rates of Volmer and Heyrovský reactions, whereas the reaction rate of the Tafel step increases, as shown in DFT-calculated Pourbaix diagrams and kinetic models^21^.

Based on the complexity of the reaction path, also the chosen number of images within the NEB method plays an important role. Too few images may miss local reaction barriers whereas too many images will significantly increase the computational effort. In order to obtain detailed information on the charge transfer within the HERs, in this study a rather high number of images is chosen to accurately illustrate the relationship between the reaction energetics and the corresponding electronic charge distribution.

### The Volmer step

In the beginning of the hydrogen evolution process, water dissociates in the Volmer step according to Eq. (6), forming a hydroxide ion and a hydrogen atom which both adsorb on the clean magnesium surface at hollow hcp sites (see Supplementary Table S1). Starting with a partially positive charge in the water molecule, the adsorption process leads to a polarity inversion of the hydrogen atom resulting in a hydride ($\hbox {H}^{-}$) on the surface.

**Figure 2:**
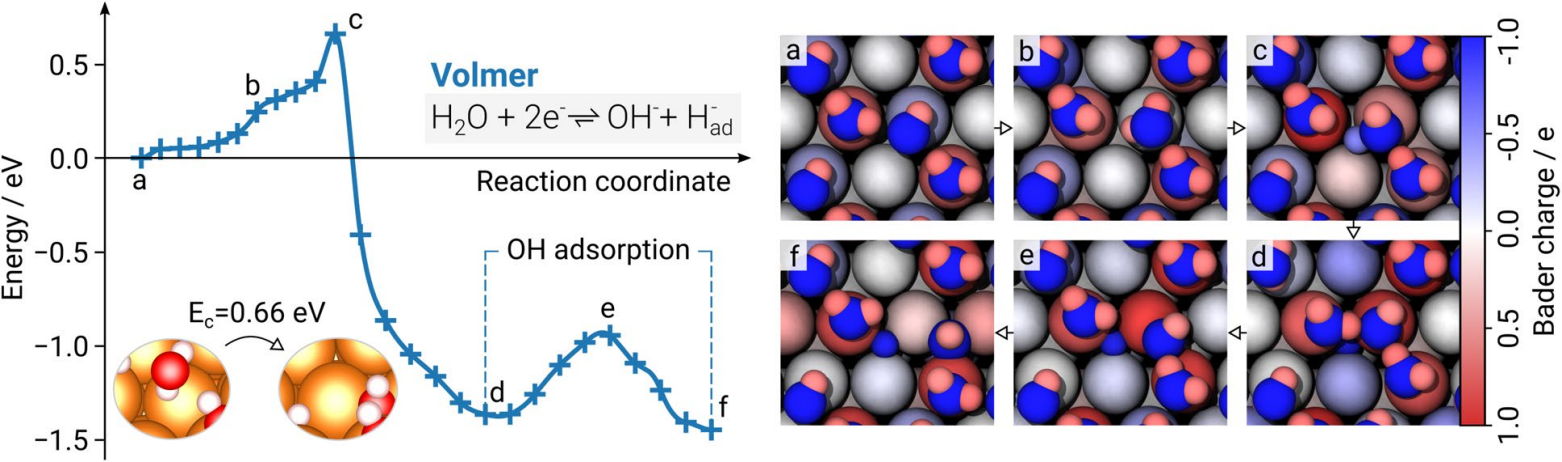
The minimum energy path (MEP) of the Volmer reaction (left) as derived from density functional theory (DFT) calculations using the climbing image nudged elastic band (NEB) method on a Mg(0001) surface. The NEB computation consists of 24 images; the MEP is presented by a force-based cubic spline. Initial and final states are illustrated along with their corresponding energy barrier $E_{\mathrm{c}}$. (a) Relaxed water bilayer. (b) Rotation of water molecule. (c) Water dissociation and adsorption of the dissociated hydrogen with a concomitant polarity inversion. (d) $\hbox {OH}^{-}$ is included in hydrogen bond network. (e) OH hydrogen bond breaks. (f) $\hbox {OH}^{-}$ adsorption at hollow hcp site. (right) Top view of the images (a–f). Atoms are colored according to their respective Bader charge in elementary charges *e*. For the sake of clarity, the representation of the charges was limited to a range between $-1$ and $1$ as the charges of atoms involved in the reaction do not exceed these values.

A water molecule in the H-down configuration is considered to dissociate in the NEB simulation as it is expected to preferentially take part in the reaction due to its small distance to the surface. In total, 24 intermediate images derived from a geometric interpolation between the assumed initial and final states sample the reaction pathway. During the NEB run, this band of images is slowly converged until it resembles the minimum energy path (MEP), as illustrated in Fig. 2. At first, the downwards-pointing water molecule is distanced around 2.3 Å from the surface with an approximate bond angle of $\phi _{{\mathrm{H}}{-}{\mathrm{O}}{-}{\mathrm{H}}}=103^\circ$ (step a). The deviation from the reference bond angle for ice $\hbox {I}_{\mathrm{h}}$ ($109.5^\circ$)^37^ indicates interactions between the Mg(0001) surface and hydrogen atoms within the adsorbed water bilayer. In contrast, the bond angle of adjacent water molecules is around $\phi_{{\mathrm{H}}{-}{\mathrm{O}}{-}{\mathrm{H}}}=109.1^\circ$ which is in good agreement with the literature value.

The interactions at the magnesium-water interface can also be observed in the electronic structure, as vividly illustrated in Bader charge analyses for the intermediate images of the NEB simulation (Fig. 2, right). Water molecules bound to top sites act here as electron acceptors, whereas those in H-down orientations act as electron donors^38^. This results in alternating negative and positive surface charges of the Mg(0001) surface, displayed as blue and red spheres, respectively. The centers of the hexagonal rings forming the water bilayer are only slightly negatively polarized. Discrete values of the charge evolution in the Volmer (as well as Tafel and Heyrovský) step are presented in Figs. S2–S4. As a reference, the Bader charges for the clean Mg(0001) surface as well as for the adsorbates of interest are illustrated in Fig. S1.

Following the MEP, the water molecule slightly rotates, positioning its downwards-pointing hydrogen atom above a hollow hcp site in preparation of the dissociation (step b). In the process, the interaction between its oxygen atom and the magnesium surface significantly increases, indicated by considerable charge fluctuations that are apparent in the surface Mg atom as well as the water molecule in its vicinity.

The point of dissociation (step c) defines the energy barrier of the reaction with $E_{\mathrm{c}}=0.66$ eV, which agrees well with previously published computational studies^24^. The water bond angle increases to $\phi_{{\mathrm{H}}{-}{\mathrm{O}}{-}{\mathrm{H}}}=110.9^\circ$ and electron densities from the surrounding Mg atoms shift to the adsorbing hydrogen atom, giving it a partially negative charge of $-0.56 \, e$ that quickly shifts to roughly $-0.95 \, e$ after completion of the water dissociation. This further increase in negative charge is caused by the remaining hydroxide ion that binds to a top site, causing an additional charge transfer from the interacting Mg surface atom to the adsorbed hydride in the process.

As hydrogen bonds form between the previously adsorbed hydroxide ion and adjacent water molecules, it is integrated in the water bilayer and a local energy minimum in the reaction is reached at $E_{\mathrm{d}}=-1.37$ eV (step d). After overcoming a local energy barrier with $\Delta E_{\mathrm{e}}=0.42$ eV (step e), the hydroxide ion adsorbs at a neighboring hollow hcp site and the global energy minimum $E_{\mathrm{f}}=-1.45$ eV of the Volmer step is attained (step f). The final charge of the adsorbed hydride was determined as $-0.96 \, e$.

**Figure 3:**
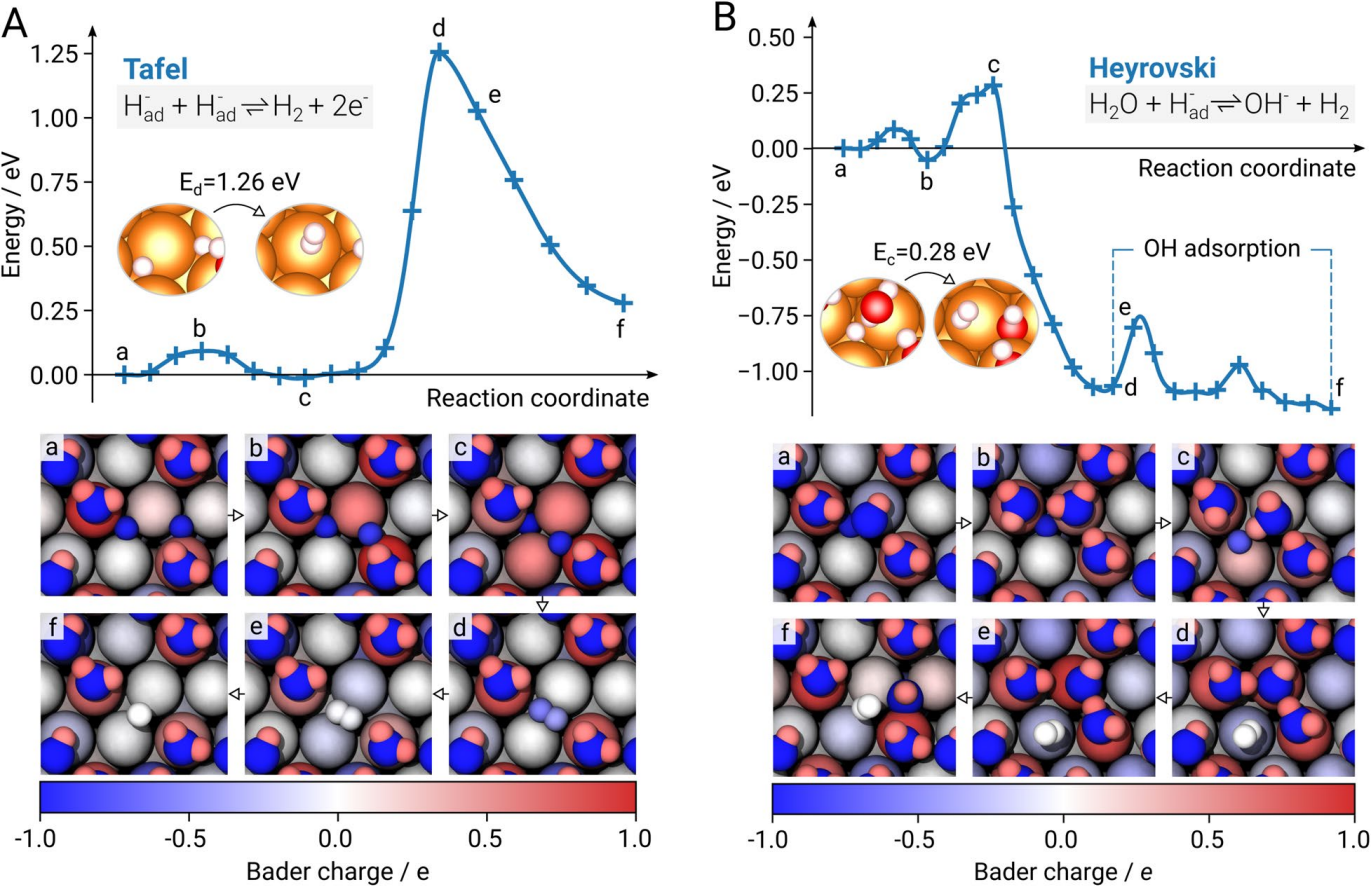
The minimum energy path (MEP) of the Tafel (**A**) and Heyrovský (**B**) reactions (top) as derived from density functional theory (DFT) calculations using the climbing image nudged elastic band (NEB) method on a Mg(0001) surface. Each NEB computation consists of 24 images; the MEP is presented by a force-based cubic spline. Initial and final states are illustrated along with their according energy barriers $E_{\mathrm{c}}$ and $E_{\mathrm{d}}$. Tafel: (a) Two adsorbed $\hbox {H}^{-}$ atoms (b) Passing bridging site (c) Adsorption at adjacent hcp and fcc sites (d) Decreasing distance at bridging site (transition state) (e) adsorbed $\hbox {H}_2$ (f) $\hbox {H}_2$ desorbs. Heyrovský: (a) Relaxed water bilayer with adsorbed $\hbox {H}^-$ (b) Rotation of $\hbox {H}_2\hbox {O}$ and surface interaction (c) Dissociation (transition state) (d) $\hbox {H}_2$ formation, integration of $\hbox {OH}^{-}$ into water bilayer (e) breaking H bond (f) OH adsorption. (bottom) Top view of the images (a-f). Atoms are colored according to their respective Bader charge. For the sake of clarity, the representation of the charges was limited to a range between − 1 and 1 as the charges of atoms involved in the reactions do not exceed these values.

### The Tafel step

**Figure 4:**
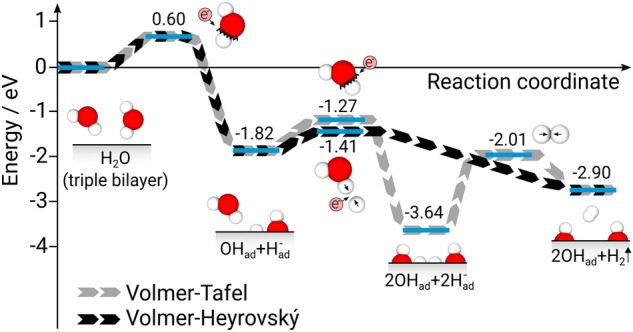
Energy barriers for the Volmer–Tafel and Volmer–Heyrovský pathways as derived from a Mg–water interface model with three water bilayers adsorbed on the clean Mg(0001) surface.

After the Volmer step the hydrogen evolution process can continue in two ways either following the Tafel pathway (Eq. (7)) or the Heyrovský reaction (Eq. (8)). During the Tafel step two adsorbed hydrogen atoms recombine which subsequently causes gas evolution after desorption of the formed $\hbox {H}_2$. The adsorption of two hydrogen atoms in neighboring fcc sites (fcc-fcc configuration) yields almost isoenergetic ($\Delta E_{\mathrm{ads}}$ = 0.05 eV) adsorption energies compared to the hcp-hcp configuration (see Supplementary Table S2). As the hydrogen atom in the Volmer step is preferentially adsorbed at hcp sites, the hcp-hcp configuration is considered as the starting point for finding the MEP. To put emphasis on the determination of the energy barrier of the hydrogen recombination on the Mg(0001) surface, influences of adsorbed hydroxide as well as the formerly reacted water molecule in the Volmer step have been neglected for the NEB simulation of the Tafel reaction pathway. An MEP including co-adsorbed hydroxide ions and additional water bilayers is illustrated in Fig. 4.

The potential reaction pathway is again sampled using 24 intermediate images between the initial and final states whereas the latter is designated as released $\hbox {H}_2$ bound to the water bilayer. While the first 17 images illustrate the reaction pathway, subsequent images merely show reorientations of the $\hbox {H}_2$ molecule and were consequently neglected in the presented MEP (Fig. 3A).

Initially the two hydrogens are adsorbed at adjacent hcp sites, both exhibiting a charge of $-0.95$ to $-0.96\,e$ (step a). Concomitantly, three out of five adjacent Mg atoms located in the top layer are positively polarized.

To initiate the Tafel reaction one hydrogen atom diffuses from its hollow hcp site across a Mg-Mg bridging site to the hollow fcp site ($E_{\mathrm{c}}=-0.01$ eV) by overcoming a local energy barrier of $\Delta E_{\mathrm{b}}=0.09$ eV. The distance between the investigated hydrogen atoms is reduced from around 2.98 Å to 2.36 Å  (step b and c) by the diffusion process. Additionally, positive polarizations of adjacent Mg atoms are shifted according to the diffusion path. The hydrogen atom at the hollow hcp site is pushed to the tetrahedral site which results in an increase of its negative charge to $-1.02 \, e$ due to the Mg atom below.

Along the MEP the atomic distance of the neighboring hydrides further decreases to 0.95 Å at the bridging site between the two atoms causing a distinct rise in energy until the reaction barrier $E_{\mathrm{d}}=1.26$ eV is reached (step d) before $\hbox {H}_2$ is formed. This is accompanied by a simultaneous charge transfer between the dissociating hydrogen and the surface Mg atoms. The computed energy barrier for the Tafel reaction is in good agreement with the literature^24^.

As the $\hbox {H}_2$ molecule diffuses to the water bilayer (step e and f), the energy of the MEP decreases until a charge neutral $\hbox {H}_2$ molecule with a final atomic distance of 0.75 Å  is attained. The final image of the MEP exhibits a relative energy of $\Delta E=0.23$ eV in comparison to the starting point (image a). The negative charge density of the hydrogen atoms is transferred to Mg surface atoms that act as binding sites of water molecules.

### The Heyrovský step

Characteristic of the Heyrovský step is that a water molecule directly reacts with an adsorbed hydrogen atom (Eq. (8)). Subsequently, $\hbox {H}_2$ is released and the remaining hydroxide ion forms a bond with the Mg(0001) surface. Here, an adsorption at a hollow hcp site is energetically favorable. The initially relaxed water bilayer is considered as starting point for finding the MEP of this reaction. Similar to the investigation of the Tafel step it is assumed that the hydride is adsorbed at a hollow hcp site at the start of the Heyrovský reaction. Moreover, the corresponding hydroxide ion is neglected here to put emphasis on the isolated reaction between $\hbox {H}_2\hbox {O}$ and $\hbox {H}_{\mathrm{ad}}$. A NEB simulation using 24 intermediate images results in the final MEP of the Heyrovský reaction, as illustrated in Fig. 3B.

The initial distance between $\hbox {H}_{\mathrm{ad}}$ and $\hbox {H}_{\mathrm{down}}$ of the reacting water molecule is comparably low with roughly 1.6 Å (step a). However, before the hydrogen atom separates, the water molecule rotates and has to overcome a small energy barrier of 0.08 eV. The reorientation results in a stronger interaction with the Mg surface and subsequently in an energetically more favorable restructuring of the water bilayer (step b). As the distance between $\hbox {H}_{\mathrm{ad}}$ and $\hbox {H}_{\mathrm{down}}$ further decreases to 1.3 Å, $\hbox {H}_{\mathrm{ad}}$ migrates over a bridging site to a near top site (step c). The intensified surface polarization facilitates the deprotonation of the water molecule and a proton transfer occurs at an energy barrier of $E_{\mathrm{c}}=0.28$ eV.

Subsequently, the images of the MEP distinctively decrease in energy to $E_{\mathrm{d}}=-1.07$ eV under formation of $\hbox {H}_2$. Simultaneously, the hydroxide ion is incorporated in the hydrogen bond network of the water bilayer (step d). In the last steps of the MEP, the hydroxide ion adopts an energetically slightly more favorable adsorption geometry ($E_{\mathrm{f}}=-1.17$ eV) through migration to a nearby hollow hcp site (step e and f) by overcoming a small energy barrier of $\Delta E_{\mathrm{e}}=0.27$ eV.

### Reaction kinetics

All energy maxima of the MEP scans for the Volmer, Tafel, and Heyrovský reactions were confirmed as transition states by vibrational analyses, showing exactly one imaginary frequency, respectively. The corresponding normal modes reflect the change in geometry in going from reactants to products. Hence, the reaction rates *k* of the HER steps can be determined by inserting the corresponding activation energies $E_{\mathrm{act}}$ into the Arrhenius equation:

10 $$\begin{aligned} k=\nu \,e^{-E_{\mathrm{act}}/k_{\mathrm{B}}\,T}. \end{aligned}$$

In the case of the Volmer step, a temperature of $T=300\,\mathrm {K}$, a calculated prefactor^39^ of $\nu =14.9\,\mathrm {THz}$ (with a reference value of $10.0\,\mathrm {THz}$)^40^, and the Boltzmann factor $k_{\mathrm{B}}$ lead to a reaction rate $k=1.1\times 10^{+2}\,\mathrm {s}^{-1}$. The exchange current density $i_0$ can be estimated^18^ by applying

11 $$\begin{aligned} i_0=k\,e\,N\,/\,A \end{aligned}$$

where $A\,/\,N$ is the surface area per Mg atom. Using the experimental value $i_{0,\mathrm {HER}}=4\times 10^{-6}\,\mathrm {A}\,{\mathrm{cm}}^{-2}$, Eqs. (10) and (11) can be employed for validation of the computed energy barriers by calculating an estimated energy barrier $E_{\mathrm{act,e}}$^18,41^. According to the previously calculated energy barriers, $E_{\mathrm{act,e}}$ as well as the reaction kinetics for all reaction steps were determined and summarized in Table 1.

**Table 1:** Activation energies $E_{\mathrm{act}}$ and estimated activation energies $E_{\mathrm{act,e}}$ for the Volmer, Tafel, and Heyrovský step at the Mg(0001) surface.

	$E_{\mathrm{act}}$/eV	$\nu$/THz	*k*/$\hbox {s}^{-1}$	$E_{\mathrm{act,e}}$/eV
Volmer	0.66	14.9	$1.1\times 10^{+2}$	0.90
Tafel	1.26	86.7	$4.8\times 10^{-8}$	0.94
Heyrovský	0.28	0.9	$1.4\times 10^{+7}$	0.82

Values for $E_{\mathrm{act},\mathrm{e}}$ are based on the experimental exchange current density $i_{0,\mathrm {HER}}=4\times 10^{-6}\,\mathrm {A}\,{\mathrm{cm}}^{-2}$, the calculated prefactor $\nu$ and the reaction rate *k*.

The estimated energy barrier $E_{\mathrm{act,e}}$ is to be understood as an upper limit for $E_{\mathrm{act}}$. As in the present model a clean Mg(0001) has been assumed but in aqueous solution most of the Mg surface is covered with MgO and Mg(OH)$_2$^14^—thus providing fewer active surface sites directly associated with HER in comparison—an effect that could reduce the estimated barrier is to include a reduced number of reactive sites in the calculation^18^. Furthermore, this anticipated surface composition corresponds to an alkaline environment, thus differing from the simulation conditions of the underlying computational model (pH 0 at OCP). As for increasing pH values the rate of the Volmer reaction is expected to decrease^21^, the associated energy barrier would increase accordingly, hence approaching the estimated energy barrier.

In this respect, the obtained energy barrier for the Volmer reaction appears to match best the estimated value derived from experiments ($\Delta E=E_{\mathrm{act,e}}-E_{\mathrm{act}}=0.24$ eV). The energy barrier for the Tafel step is significantly larger than the upper limit $E_{\mathrm{act,e}}$ ($\Delta E=-0.32$ eV) and results in a relatively low reaction rate *k* in the order of $10^{-8}\,\mathrm {s}^{-1}$. For the Heyrovský step, the calculated energy barrier is significantly lower ($\Delta E=0.54$ eV), resulting in an accordingly high reaction rate *k* in the order of $10^{+7}\,\mathrm {s}^{-1}$. Based on these results, it becomes evident that the Volmer step is the rate-limiting step for HER at the Mg(0001) surface, rather than the Tafel step as previously assumed^19^. Adsorbed hydrogen due to the Volmer step would react in high rates with interacting water molecules (Heyrovský step) leading to hydrogen evolution, whereas the contribution of recombining hydrogen atoms (Tafel step) is rather small.

After the isolated investigation of the dominant reaction mechanisms in the hydrogen evolution process, further extensions to the model are supposed to quantify the impact of the simplifications made beforehand. Hence, two additional explicit water bilayers as well as intermediate reaction products (consisting of adsorbed OH ions) are included in order to obtain a comprehensive understanding of the energy barriers in the Volmer–Tafel and Volmer–Heyrovský pathways. The results are illustrated in Fig. 4.

Although additional water bilayers as well as the inclusion of intermediate reaction products have an impact on the occurring energy barriers, the outcome of the Volmer–Heyrovský pathway being the energetically most favorable reaction pathway for the HER on Mg remains unaltered. On the one hand, this confirms results by previous studies^20–22,26^, whereas on the other hand, the model applied here provides valuable additions which allow a deeper insight into the charge migration within the occurring reaction mechanisms.

### Magnesium dissolution

Analogously to the cathodic HER, the anodic reaction in aqueous Mg corrosion is defined by magnesium dissolution as described in Eq. (2). In that process, Mg dissociates from the surface, undergoing oxidation to $\hbox {Mg}^{2+}$. A study by Petty in 1954^42^ proposed Mg could also be dissolved as monovalent $\hbox {Mg}^{+}$, giving rise to a popular theory that HE on dissolving Mg surfaces arises from a secondary reaction between $\hbox {Mg}^{+}$ and water where additional hydrogen gas is formed. Although there is no persuasive experimental evidence, this theory is largely promoted in particular as an explanation for the increased HE rates during anodic dissolution (negative difference effect / anodic HE)^14,33,34^. Aside from other popular theories trying to explain this phenomenon^14,33,34^, recent experimental findings suggest an enhanced catalytic activity of magnesium primarily associated with regions dominated by the anodic dissolution reaction^31,41,43^. These findings are supported by a theoretical study, where HE rates increase with simultaneous dissolution of Mg for increasing potentials^22^. Taylor et al. give a possible explanation for this behavior by suggesting that parts of the hydroxide (passivation) layers are removed anodically through the dissolution of the Mg atom to which the OH is adsorbed. In this way, more clean surface area would be revealed, thus further promoting water dissociation and hydrogen evolution^44^.

Based on the reported experimental and theoretical findings, two potential reaction pathways are revealed, where the dissolving magnesium atom is either exclusively assisted by water or co-adsorbed hydroxide ions further facilitate the dissolution^21^. Due to limitations of our employed computational model, the focus in this study shall lie on the water-assisted reaction pathway.

**Figure 5:**
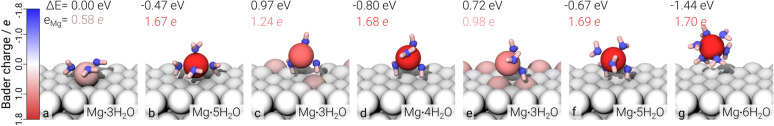
Characteristic images of the reaction pathway of a dissolving magnesium ion. Only the first solvation shell of the Mg ion is illustrated. Values for the relative energies $\Delta E$ and Bader charges $\mathrm {e}_{\mathrm{Mg}}$ are depicted in the upper left corner of each image. The atoms are colored according to their respective Bader charges.

A NEB calculation has been set up to gain a deeper understanding of the reaction kinetics as well as the charge evolution of the dissolved Mg ion. The computational model used so far in this work has been further extended to include an additional Mg atom adsorbed upon the Mg(0001) surface, thus representing a more realistic, rougher Mg surface. Additionally, a total number of four water bilayers have been included to ensure sufficient water molecules for full solvation of the dissolving Mg ion. The described extended model acts as initial state of the NEB calculation. For the final state, the adsorbed Mg atom is moved away from the surface into solution before it is relaxed, resulting in a solvation shell of six water molecules in an octahedral arrangement, agreeing well with the literature^45^. The model is illustrated in Fig. S5. As the water structure shifts during dissolution, the NEB path is dominated by relatively large energy fluctuations, hampering its convergence. Hence, for a higher resolution of the reaction pathway the number of intermediate images was further increased to 48. In order to gain a deeper understanding of the dissolution pathway and the involved charge evolution, the relaxed MEP and corresponding Bader charges of the dissolving Mg ion are presented in Fig. S6. Characteristic images are visualized in Fig. 5 in more detail.

During the dissolution the Mg ion passes a number of favorable coordination states which entail changes in the energetic states and charge distribution. While adsorbed on the Mg(0001) surface, the Mg atom is coordinated by three water molecules with a Bader charge of $0.58\,e$ (step a). After overcoming an initial energy barrier of $\Delta E=0.44$ eV, the Mg atom is ionized (step b). More water molecules are bound to the Mg ion increasing its coordination number to five, its Bader charge to $1.67\,e$, and its distance from the surface by around 1.55 Å to 3.99 Å– marking the transition to the dissolved state. During the remaining observed dissolution process (steps c-g), the coordination number of the Mg ion varies between three and six water molecules. For a coordination with three water molecules (step c and e), the Mg ion shows again interaction with the surface and actually exhibits a Bader charge of around $1\,e$ indicating an existing $\hbox {Mg}^{+}$ species. However, investigating the MEP reveals that Mg$\cdot$3$\hbox {H}_2$O complexes can only be found within high energy barriers (0.97 eV) and are thus energetically highly unfavorable. For higher coordination number (four to six water molecules) the Bader charge of the Mg ion becomes stable at a value of around $1.7\,e$, confirming $\hbox {Mg}^{2+}$ as the energetically most favorable charge state during Mg dissolution. After formation of the $\hbox {Mg}^{2+}\cdot \hbox {6H}_2\hbox {O}$ complex, the relative energy reaches a minimum at $\Delta E=-1.44$ eV.

Again, an estimate for the energy barrier of Mg dissolution can be derived from experimental results according to Eqs. (10) and (11). Assuming a prefactor $\nu =1\times 10^{13}$ Hz and an anodic exchange current density $i_{0,\mathrm {Mg}}=1.6\times 10^{-5}\,\mathrm {A}\,{\mathrm{cm}}^{-2}$, the reaction barrier can be estimated to 0.85 eV^40,46^. Comparison with the calculated value of 0.97 eV indicates that the dissolution reaction is not solely water-assisted but rather facilitated by co-adsorbed species, for instance hydroxide ions, as already proposed in the literature^21,22,44^. DFT calculations for the solvated $\hbox {Mg}^{2+}$ ion and $\hbox {Mg(OH)}_2$ molecule provide further evidence for this indication (Fig. S7). Although the combination of the NEB method and Bader charge analysis results in a deep insight into the minimum energy path of a dissolving Mg ion, the continuous shifts in the water structure require a high resolution of the reaction pathway, resulting in an immense computational cost. For more clarity, further studies employing dynamic simulation methods, such as metadynamics-based *ab initio* molecular dynamics simulations, would be beneficial to cross-check the results, to study co-adsorption explicitly and to yield additional insights. Yet, the obtained results vividly illustrate the energy barriers and charge evolution of an Mg ion undergoing dissolution and strongly contradict the claim of a energetically stable $\hbox {Mg}^{+}$ species which promotes water reduction.

## Discussion

This study provides a deeper mechanistic understanding of the hydrogen evolution on the Mg(0001) surface based on density functional theory (DFT) calculations. The two predominant hydrogen evolution reaction (HER) pathways, the Tafel and Heyrovský reaction, during the corrosion process of Mg have been investigated using the climbing image nudged elastic band (NEB) method in combination with concurrent Bader charge analyses of each NEB image. Prerequisite to the two reaction schemes considered in this study is the dissociation of a water molecule in the Volmer step which leads to adsorption of a hydroxide and a hydride ($\hbox {H}^{-}$) on the Mg surface. The results of the NEB calculations indicate that the energy barrier for the subsequent formation of hydrogen is lower for the Heyrovský pathway than for the Tafel step, confirming the Volmer–Heyrovský reaction cascade as the predominant mechanism that leads to the formation of hydrogen during the degradation of magnesium. Furthermore, the comprehensive analysis of the occurring energy barriers revealed that the Volmer reaction is the rate-limiting step of the HER.

Additionally, the charge state and energy barriers of an Mg ion detaching from the bulk material during the dissolution of magnesium was investigated in detail employing the same method as for the HER. The obtained results indicate involvement of co-adsorbed species. Concomitantly, the findings strongly contradict the existence of a stable $\hbox {Mg}^{+}$ species involved in the water dissociation process, a mechanistic detail which was controversially discussed in the literature over the course of the last decade.

Particularly Mg corrosion engineering depends on a deep understanding of the underlying processes. The results presented in this study coupled with the detailed validation of recent claims provide an insightful contribution to the realm of Mg corrosion research and can inspire novel approaches to gain control over the degradation behavior of Mg-based materials.

## Methods

### Computational details

The results presented in this study were obtained from density functional theory (DFT) calculations using the plane-wave (PW) code Vienna Ab Initio Simulation Package (VASP)^47–50^ with the projector augmented-wave (PAW) method^51,52^. Since van der Waals (vdW) interactions are a key factor in hydrogen evolution reactions (HERs)^53^, the exchange-correlation (XC) functional optB88-vdW^54–59^ was employed for all DFT computations. This functional accounts for dispersion interactions in an approximate fashion by including a non-local correlation part in the exchange-correlation energy and is expected to capture well the subtle energetic contributions of hydrogen bonded systems in solid–liquid interfaces^53,60,61^.

All simulations were performed using a $6\times 6\times 1$ gamma-centered grid of k-points^62^. The plane-wave expansion was limited by a cutoff energy of 520 eV. During the relaxation process, the atom positions were allowed to adjust until the atomic forces were less than 5 meV Å$^{-1}$. Subsequently, the structure was investigated statically to obtain more accurate total energies via the tetrahedron method with Blöchl corrections^63^.

The Mg(0001) surface was modeled using a slab of five Mg layers in a $3\times 3$ surface cell. Atoms in the two lowermost layers were kept fixed in their bulk-like positions, whereas the remaining layers were free to relax, thus being able to respond to occurring forces due to surface effects or adsorption. Relaxations of the bulk resulted in lattice constants $a=3.19$ Å and $c=5.15$ Å, which are in good agreement with theoretical and experimental values in literature^20,23,64^. The solid–liquid interface was modeled by adding (at least) one bilayer of water consisting of six explicit water molecules. A vacuum region of around 20 Å was added above the water bilayer to avoid interaction between the periodic images. Additionally, a dipole correction^65^ was applied to compensate for the slab asymmetry.

### Charge evolution simulations

Identifying energy barriers in potential corrosion mechanisms increases the understanding of reaction kinetics and helps to dismiss unlikely reaction pathways from corrosion models. Furthermore, as can be seen in the contemplable reaction pathways in Mg corrosion, electron transfers play a significant role and also contribute to the reaction energetics. Hence, to simulate the minimum energy paths (MEPs) and occurring charge transfers in potential hydrogen evolution pathways as well as Mg dissolution reactions, we combined two methods in this study: the climbing image nudged elastic band method and the Bader charge analysis.

The climbing image nudged elastic band^66,67^ (CI-NEB, in this study referred to as NEB) method allows finding saddle points and minimum energy paths (MEP) between known reactants and products. Interpolation between the initial and final states of the expected reaction gives a number of intermediate images along the reaction coordinate which are then being optimized by minimizing the acting forces. Each image is relaxed to the lowest energy possible while keeping equal spacing to adjacent images. Convergence of the band was assumed when the computed forces fell below 0.05 eV Å$^{-1}$.

The Bader charge analysis^68–70^ allows dividing a molecule into non-overlapping atomic domains with well-defined boundaries (Bader volumes) by analyzing the electronic charge density. Henkelman et al. developed an efficient way to partition a charge density grid derived from PW-DFT calculations into Bader volumes^70^. Their implementation uses steepest ascent paths along the charge density gradient from grid point to grid point until a charge density maximum is reached. The charges encased in the resulting Bader volumes are a good approximation for the actual charge state of an atom.

Introducing a novel approach of combining the two aforementioned methods it is possible to simulate and visualize the charge evolution along a reaction pathway, thus indicating potential relationships between occurring charge transfers and reaction energetics.

## Supplementary information

Supplementary Information.

## Data Availability

The authors declare that the main data supporting the findings of this study are available within the paper and its supplementary files. The code for the visualization of the charge evolution is deposited on GitHub (https://github.com/koerper/baderVis). Other relevant data are available from the corresponding author upon reasonable request.
