# Supplementary material for: A first-principles analysis of the charge transfer in magnesium corrosion
Source: Sci Rep. 2020 Sep 14;10:15006. doi: 10.1038/s41598-020-71694-4 (PMC7490698; doi:10.1038/s41598-020-71694-4)
Supplement: Supplementary file 1 — Supplementary Information. [file 41598_2020_71694_MOESM1_ESM.pdf]

Supplementary Information: A First-Principles Analysis of the Charge Transfer in Magnesium Corrosion

Tim Würger,[†,‡] Christian Feiler,[†] Gregor B. Vonbun-Feldbauer,[¶] Mikhail L. Zheludkevich,[†,§] and Robert H. Meißner[*,†,‡]

†*Institute of Materials Research, Helmholtz-Zentrum Geesthacht Zentrum für Material-und Küstenforschung GmbH, Geesthacht, Germany*

‡*Institute of Polymer and Composites, Hamburg University of Technology, Hamburg, Germany*

¶*Institute of Advanced Ceramics, Hamburg University of Technology, Hamburg, Germany*

§*Institute for Materials Science, Faculty of Engineering, University of Kiel, Kiel, Germany*

E-mail: robert.meissner@tuhh.de

Table 1: Adsorption energies (in eV) of H_2O, OH and H at potential adsorption sites on the Mg(0001) with respect to the employed exchange-correlation functional. Dashes ("—") denote energetically unfavorable states which relaxed to other adsorption sites. Results were obtained from *in vacuo* simulations. Starred values were derived from calculations involving one water bilayer.

Functional	Adatom	top	bridge	fcc	hcp
PBE	H_2O	−0.42	—	—	—
	OH	—	—	−5.32	−5.35
	H	—	—	−0.05	−0.04
optB88-vdW	H_2O	−0.50 / −0.59*	—	—	—
	OH	—	—	−5.50 / −5.44*	−5.53 / −5.45*
	H	—	—	−0.10 / −0.15*	−0.09 / −0.12*

Table 2: Reaction energies (in eV) of the Volmer and Tafel step for different OH and H adsorption sites on Mg(0001) with respect to the inclusion of solvent (one water bilayer) in the simulation.

	Reaction	fcc+fcc	fcc+hcp	hcp+fcc	hcp+hcp
Vacuum	$H_2O \rightarrow OH_{ad}+H_{ad}$	-1.67	-1.60	-1.49	-1.68
	$H_{ad}+H_{ad} \rightarrow H_2$	0.25	0.18	0.18	0.20
Water bilayer	$H_2O \rightarrow OH_{ad}+H_{ad}$	-1.43	-1.22	-1.40	-1.45
	$H_{ad}+H_{ad} \rightarrow H_2$	0.28	0.25	0.25	0.23

Table 3: Reaction energies E_r and barriers E_b for the Volmer, Tafel and Heyrovský step in the hydrogen evolution process on the Mg(0001) surface with respect to the number of explicitly modeled water bilayers. For the *in vacuo* case no energy barriers were determined.

	Volmer			Tafel			Heyrovský		
# Water bilayers	0	1	3	0	1	3	0	1	3
Reaction energy E_r	-1.68	-1.45	-1.82	0.20	0.23	0.76	-1.36	-1.17	-1.05
Energy barrier E_b	—	0.66	0.55	—	1.27	1.63	—	0.28	0.41

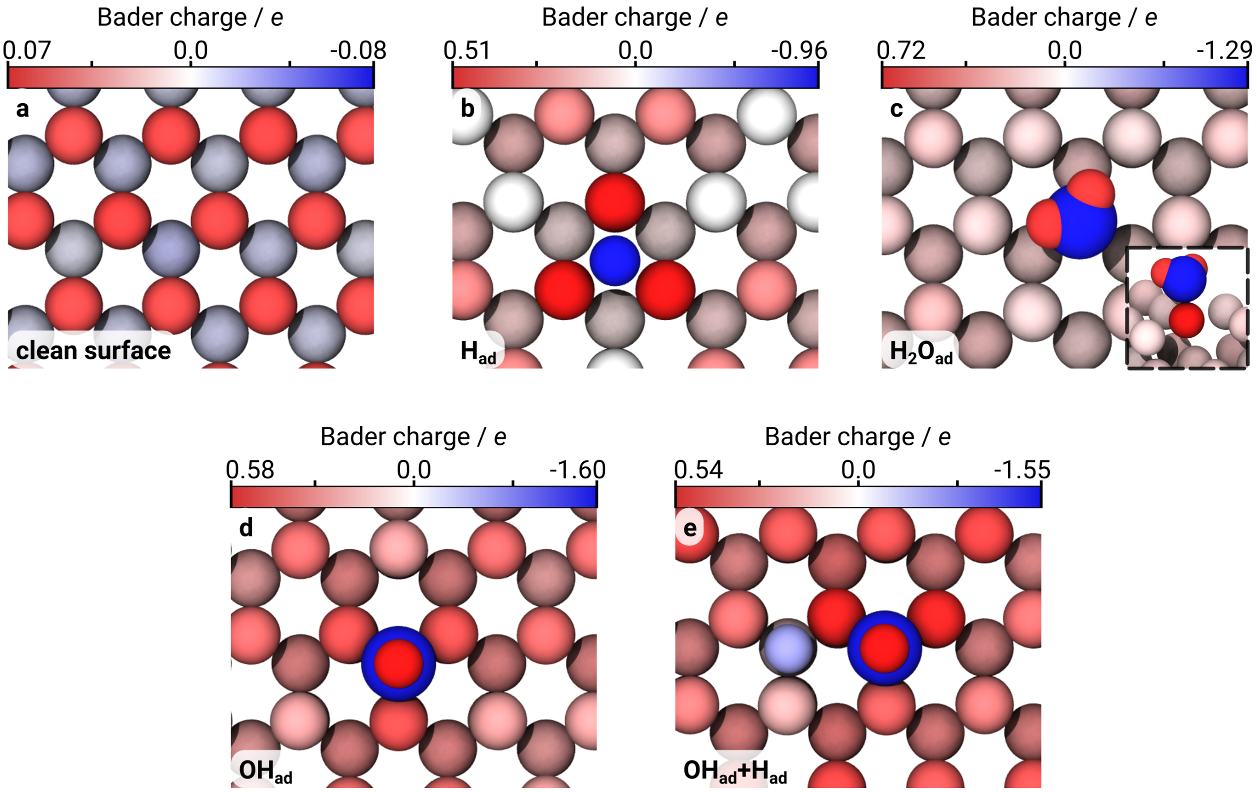

Figure S1: Bader charges of the (a) clean Mg(0001) surface, (b) adsorbed hydrogen, (c) adsorbed water, (d) adsorbed hydroxide and (e) adsorbed hydroxide and hydrogen.

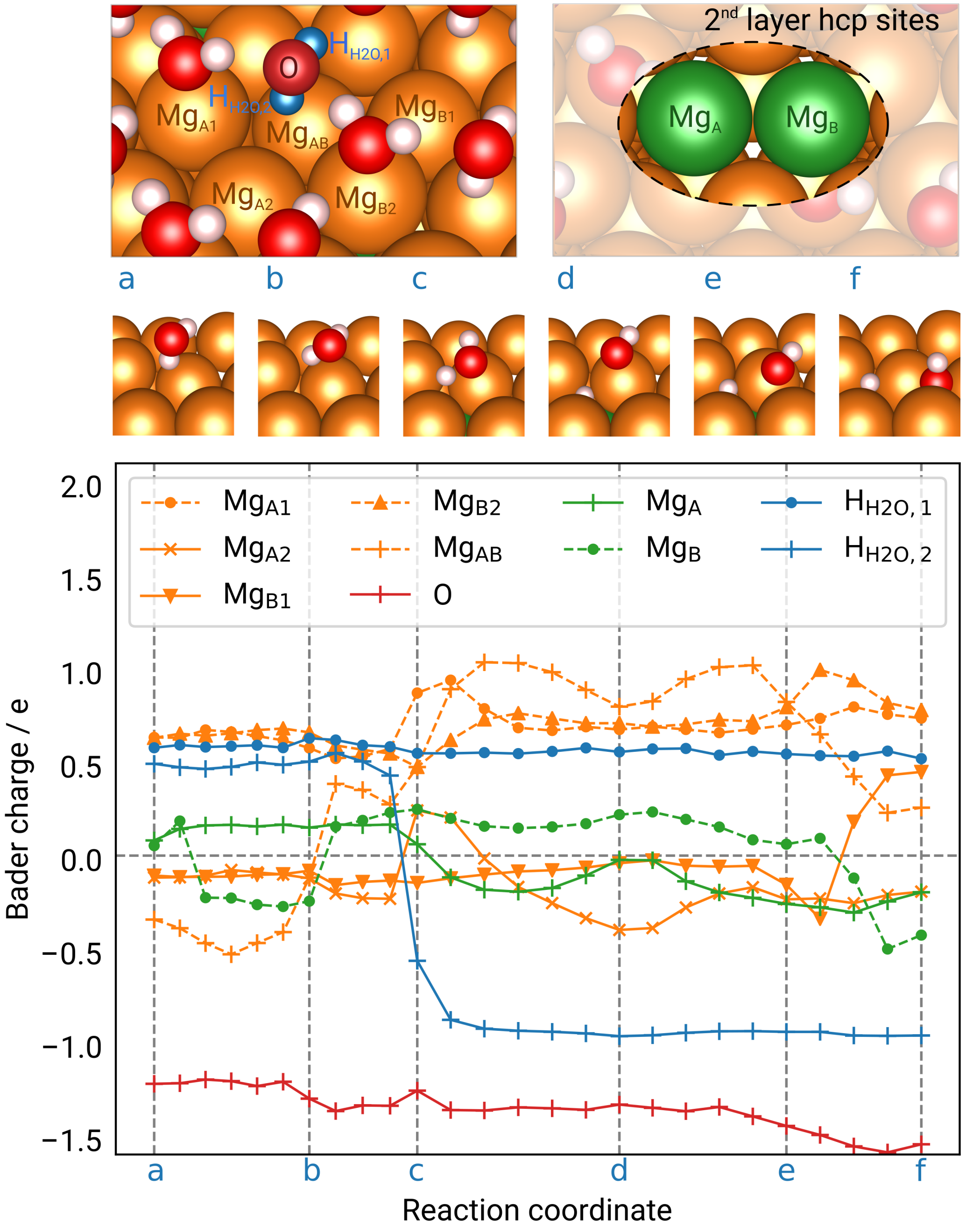

Figure S2: Bader charge evolution during the NEB simulation of the Volmer step (including one water bilayer). Characteristic reaction steps (a-f) were visualized without the surrounding water bilayer. Atoms which appear to take part in the reaction were labeled, and their Bader charge was plotted with respect to the reaction coordinate.

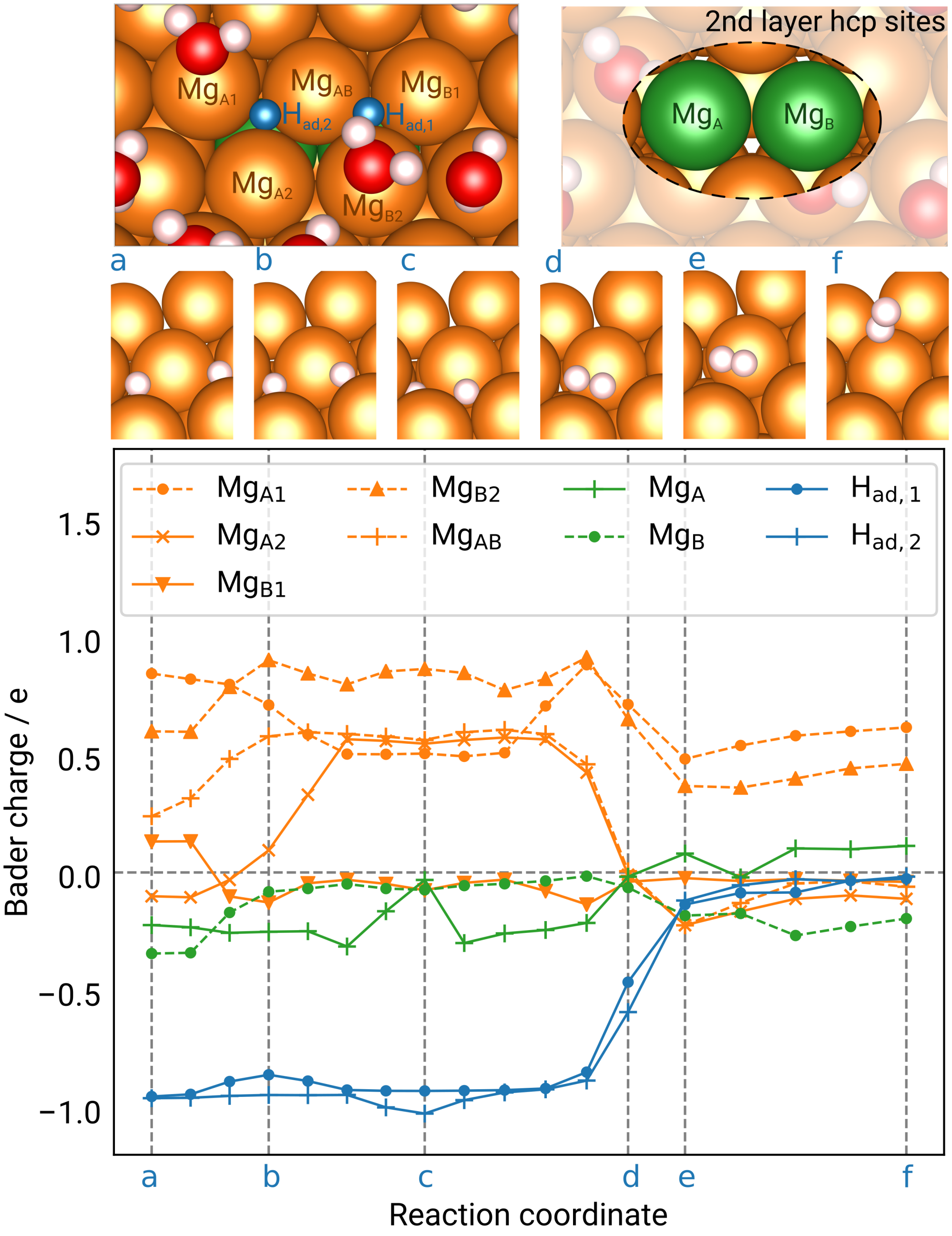

Figure S3: Bader charge evolution during the NEB simulation of the Tafel step (including one water bilayer). Characteristic reaction steps (a-f) were visualized without the surrounding water bilayer. Atoms which appear to take part in the reaction were labeled, and their Bader charge was plotted with respect to the reaction coordinate.

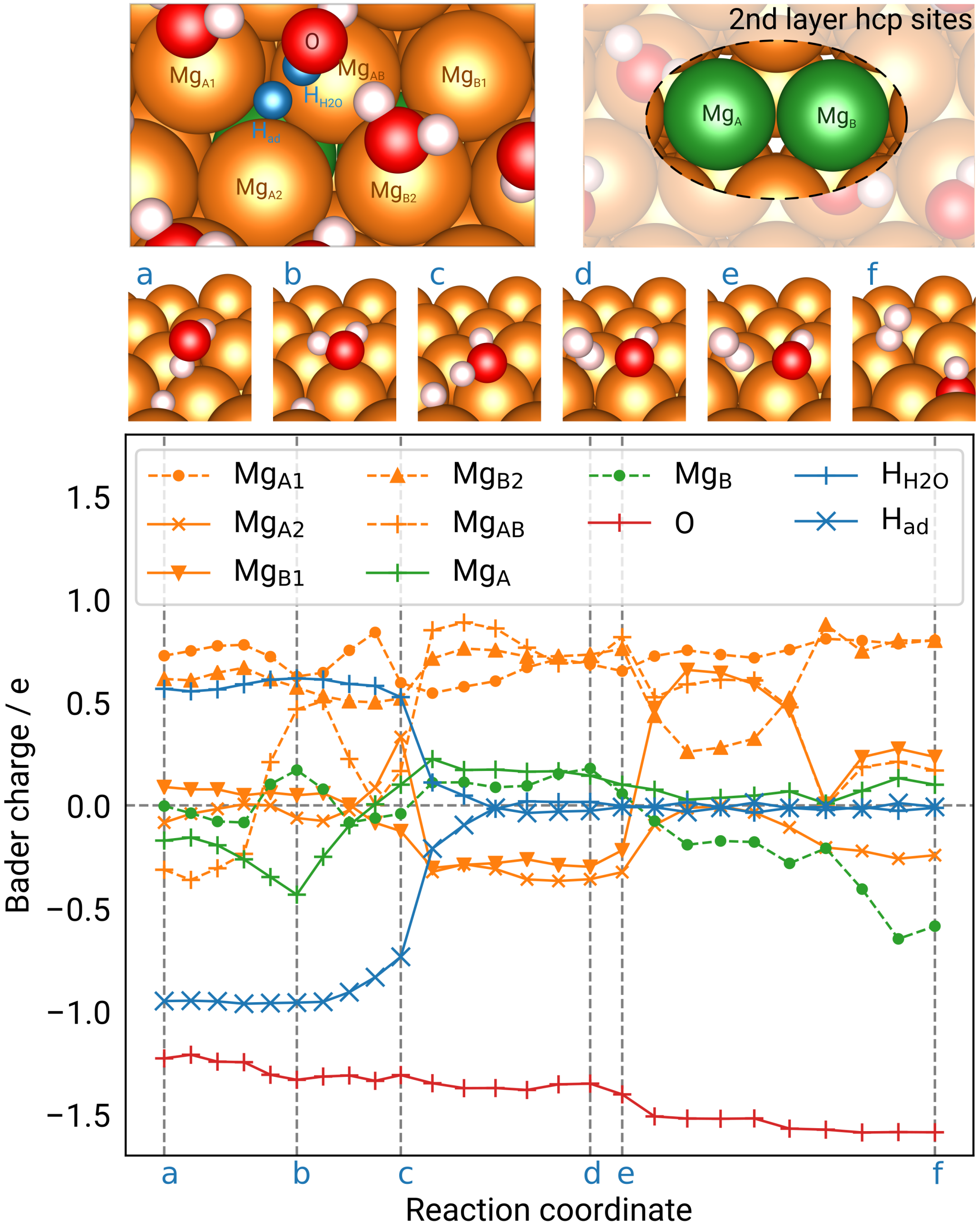

Figure S4: Bader charge evolution during the NEB simulation of the Heyrovský step (including one water bilayer). Characteristic reaction steps (a-f) were visualized without the surrounding water bilayer. Atoms which appear to take part in the reaction were labeled, and their Bader charge was plotted with respect to the reaction coordinate.

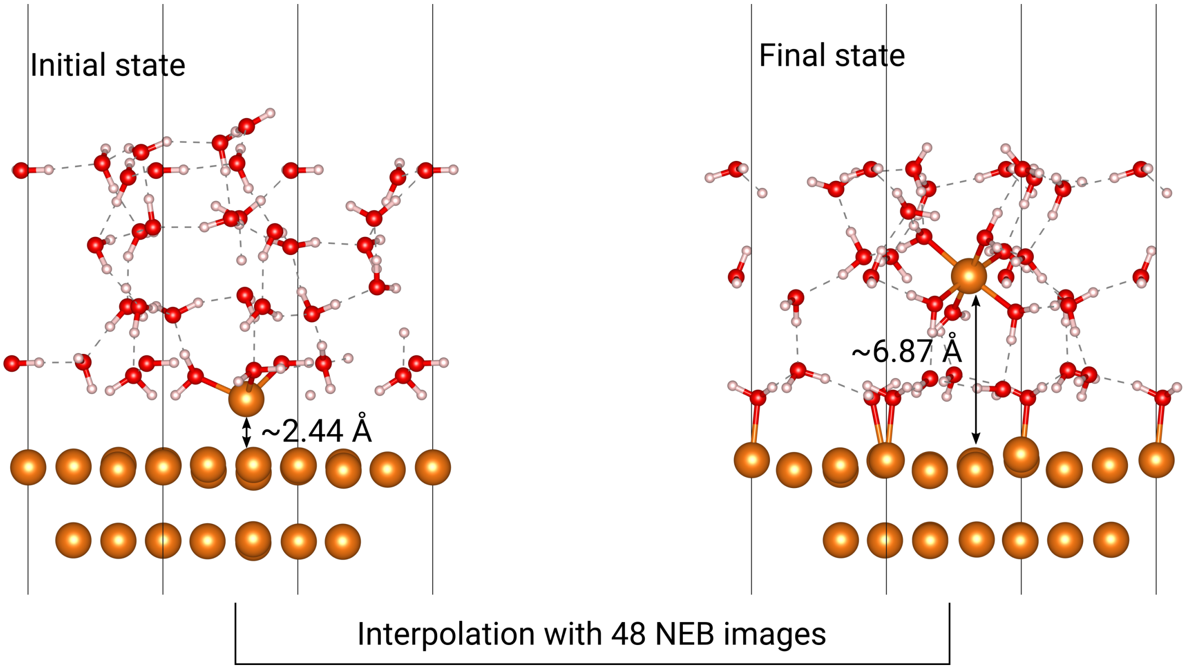

Figure S5: Mg dissolution model. The system includes a 3x3 Mg slab with five layers and four water bilayers consisting of 24 water molecules to ensure solvation of the Mg ion at all times. For creating the initial and final state for the NEB computation, one additional Mg atom was firstly placed 3 Å and 7.5 Åaway from the topmost Mg layer, respectively. Both structures were relaxed until the atomic forces were less than 5 meV $Å^{-1}$. After convergence, the relaxed structure for the final state should represent an Mg ion surrounded by six water molecules. If this was not the case, the relaxation reached a local minimum. Then, the position of the Mg ion was slightly shifted and the relaxation process was repeated. The final distances from the surface are around 2.5 Å and 6.9 Å for initial and final state, respectively. Once two well-relaxed initial and final states were obtained, the NEB computation was started. Here, a high number of 48 intermediate images is chosen in order to compensate for the energy fluctuations due to shifts in the water structure during the relaxation process.

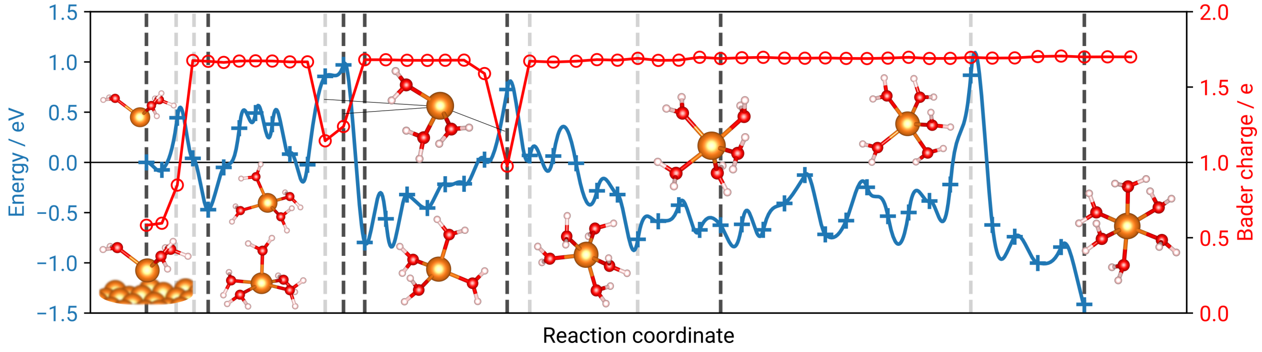

Figure S6: Minimum energy path and Bader charge evolution of the Mg dissolution reaction (including four water bilayers). Characteristic reaction steps were visualized including the first solvation shell of the Mg ion. The Bader charge of the dissolving Mg ion was plotted with respect to the reaction coordinate. The majority of the computed forces in the band fell below $0.3\,\mathrm{eV\,\AA^{-1}}$

.

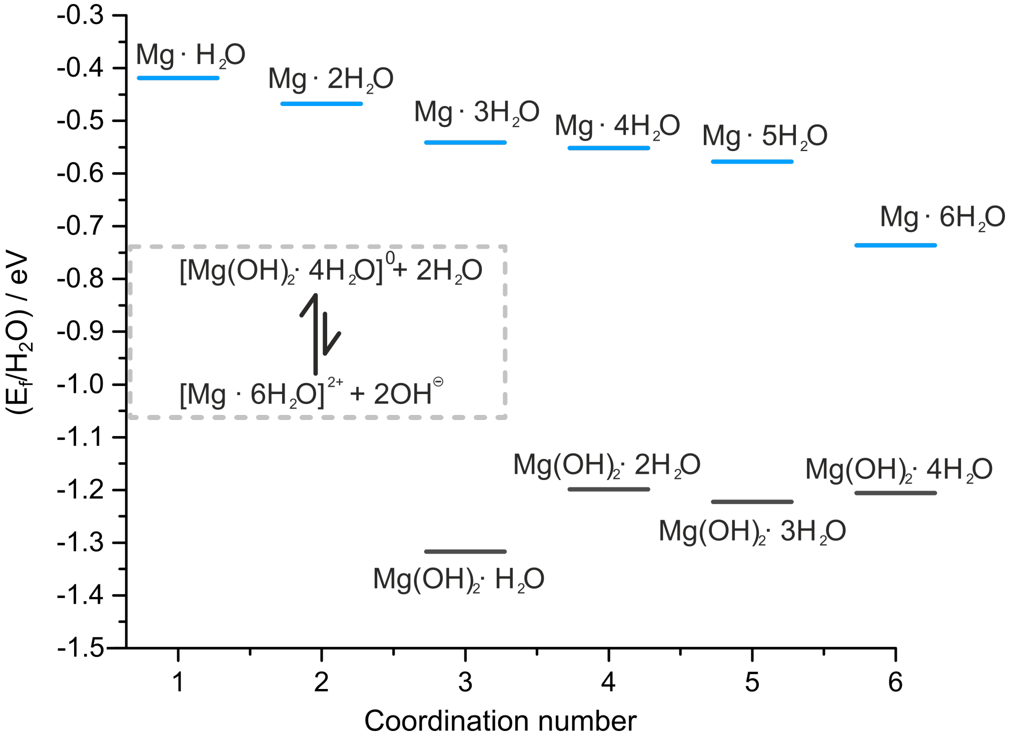

Figure S7: The complexes of Mg with water were optimized using Gaussian 16 at the TPSSh/def2SVP level of theory including Grimme D3 dispersion correction. The depicted complex formation energies of Mg(0) and $Mg(OH)_2$ with water are scaled on one water molecule. The driving force for the formation of $Mg(OH)_2$ during the dissolution of Mg is shown in the inlay (grey dashed box). The complex of $Mg(OH)_2$ with four water molecules and two water molecules in its vicinity is energetically highly favorable compared to a hexaaqua-Mg(II) complex with two neighboring hydroxide ions.
